# Functional heterogeneity of γδ T cells in colorectal cancer

**DOI:** 10.3389/fimmu.2026.1761668

**Published:** 2026-03-24

**Authors:** André Miguel Vaz-Pinto, Immo Prinz

**Affiliations:** Institute of Systems Immunology, Hamburg Center for Translational Immunology, University Medical Center Hamburg-Eppendorf, Hamburg, Germany

**Keywords:** colorectal cancer, IFN-γ-producing γδ T Cells, IL-17-producing γδ T cells, immunotherapy, tumor immunity, γδ T cells

## Abstract

Colorectal cancer (CRC) remains a significant global health concern. Improving the efficacy of immunotherapy, particularly for microsatellite-stable tumors, requires a better understanding of the unconventional T-cell populations that regulate intestinal immunity. γδ T cells are uniquely positioned at the epithelial barrier and function as rapid sentinels, recognizing stress signals independently of classical antigen presentation. In a healthy colon, γδ intraepithelial lymphocytes (IELs) and lamina propria lymphocytes (LPLs) form separate compartments influenced by tissue-specific butyrophilin-like (BTNL) interactions, microbiota-derived signals, and cytokine environments. These signals imprint divergent effector programs, ranging from IFN-γ-producing, cytotoxic responses to IL-17-driven tissue repair and inflammation. In CRC, however, these subsets exhibit remarkable plasticity. In mouse models, for example, Vγ1^+^ and Vγ7^+^ IELs mediate potent antitumor immunity, whereas Vγ4^+^ and Vγ6^+^ LPLs can acquire IL-17-dependent pro-tumor functions. In contrast, human data depict a different balance. Across multiple cohorts, tumor-infiltrating γδ T cells, predominantly the Vδ1^+^ and Vδ2^+^ subsets, exhibit robust cytotoxic and IFN-γ-associated phenotypes. Meanwhile, the existence of bona fide IL-17-producing γδ T cells remains highly controversial. Higher γδ T-cell abundance correlates with better outcomes, even in tumors with defective HLA class I expression, where γδ T cells can mediate the therapeutic effects of PD-1 blockade. Emerging findings reveal subset heterogeneity, context-dependent functional states, and a crucial role for NK-receptor-mediated recognition, particularly via NKG2D. Together, these insights position γδ T cells as pivotal yet understudied regulators of CRC progression and immunotherapy responsiveness. Understanding their subset-specific biology could lead to next-generation γδ-based therapies tailored to the unique immunogenic constraints of colorectal cancer.

## 1 Introduction

### 1.1 Colorectal cancer

Colorectal cancer (CRC) is the third most commonly diagnosed malignancy worldwide, accounting for about 10% of all cancer cases and representing the second leading cause of cancer-related deaths, particularly among individuals over 50 years of age. Its incidence and mortality vary globally, with Europe, especially Eastern Europe, among the most affected regions. Although advances in screening, surgery, and systemic treatments have improved outcomes, the global burden of CRC is still projected to rise by more than 60% by 2040, reaching approximately 3.2 million new cases and 1.6 million deaths annually (1).

Multiple factors contribute to CRC development, including age, genetic predisposition, and lifestyle behaviors such as poor diet, physical inactivity, smoking, and alcohol consumption. While preventive and early detection strategies remain crucial, therapeutic improvement is essential for patients with advanced disease. Current systemic treatments, including chemotherapy, targeted therapy, and more recently immunotherapy, have provided meaningful progress, but not in all cases. In particular, immune checkpoint blockade has demonstrated remarkable and durable efficacy in a subset of patients with mismatch repair-deficient (dMMR) or microsatellite instability-high (MSI-H) tumors, yet the majority of CRCs are microsatellite stable (MSS) and derive little to no benefit from these approaches (2, 3). These features determine how tumors interact with the immune system and influence therapeutic responsiveness. MSI-H tumors are typically infiltrated by activated cytotoxic T cells and show strong responses to checkpoint inhibitors, whereas MSS tumors often exhibit a suppressive microenvironment that excludes immune cells or impedes effective T cell activity. Understanding the mechanisms that regulate T cell infiltration, activation, and dysfunction within the CRC microenvironment is therefore critical to extending the benefits of immunotherapy to a broader group of patients and developing more effective T cell-based treatment strategies (4, 5).

### 1.2 γδ T cells of the colon

To overcome the barriers to successful anti-CRC immunotherapy, it may be useful to move beyond conventional T-cell-centered research and consider other subsets that reside in colonic tissue. In this regard, γδ T cells are particularly relevant, as they are specifically enriched at barrier sites such as the colonic mucosa and are well equipped for epithelial surveillance and detecting transformed cells. Consistent with this role, they can represent up to 50% of the resident lymphocyte compartment in the mouse small intestine and up to 20% in the mouse and human colon (6–8).

Within the colon, γδ T cells comprise two main subsets: the intraepithelial lymphocytes (IELs), which patrol between epithelial cells, and the lamina propria lymphocytes (LPLs), located deeper in the tissue (9). This compartmentalization aligns with their functions: IELs rapidly detect stressed or infected epithelial cells, striving to maintain homeostasis. At the same time, LPLs respond to infiltrating microbial metabolites and express cytokines that strengthen the tight junctions or control microbial dissemination. The TCRs of these cells likely support their functions, because the specific pairing of gamma and delta chains in mucosal γδ T cells is strongly tied to their localization and function (10).

### 1.3 γδ intraepithelial lymphocytes

In the mouse colon mucosa, γδ IELs predominantly express the Vγ7 chain. Vγ7 enables recognition of butyrophilin-like (BTNL) BTNL1, which dimerizes with BTNL6 and BTNL4. These BTNL heterodimers are uniquely expressed in the intestinal epithelium and are thought to direct the residency of Vγ7^+^ γδ IELs exclusively to this tissue (11) (**Table 1**). BTNL1 and 6 help activate the effector program of the “naïve” γδ T cells that leave the thymus and migrate to peripheral tissues. In mice, the absence of BTNL1 leads specifically to the almost complete loss of Vγ7^+^ intraepithelial lymphocytes (IELs) (11, 32).

**Table 1:** Functional and phenotypic overview of mouse and human colonic γδ T cell subsets.

Subset mice	Phenotype	Tissue/Origin	Key markers	Key transcripts	Cancer role (CRC)	References
Vγ4/Vγ6 (γδT17)	IL-17–producing; OXPHOS; microbiota-dependent	Colon lamina propria	CD44^hi^, CCR6^+^, PD-1^+^, Sca1^+^	*Rorc, Il17a, Il17f, Il23r, Il1r1, Ccr6*	Pro-tumoral (angiogenesis, neutrophil recruitment, MDSC expansion)	(9, 12, 13, 15, 16)
Vγ7/Vγ1 (IEL-type)	Cytotoxic; granzyme-rich; glycolytic; IFN-γ capable	Intestinal epithelium (IELs) -Recognize Btnl1/6.	CD8αα^+^, CD103^+^, CD107a^+^	*Gzmb, Gzma, Prf1, Ifng, Lgals3, Lag3*	Anti-tumoral (removal of dysplastic epithelial cells)	(11, 17–20)
Subset human	Phenotype	Tissue/Origin	Key markers	Key transcripts	Cancer role (CRC)	References
Vγ9Vδ2	Highly cytotoxic; IFN-γ^+^; glycolytic; phosphoantigen-responsive	Blood; lymphoid tissues	TCRVγ9^+^/Vδ2^+^, NKG2D^+^, DNAM-1^+^, CCR5^+^	*GZMB, PRF1, IFNG, TBX21, RUNX3*	Vastly anti-tumoral	(21–25)
Vδ1	Tissue-resident; stress-sensing; cytotoxic	Intestinal epithelium; tumors. Recognize BTNL3/8	CD103^+^, CD69^+^, NKG2D^+^, PD-1^+^. Most commonly paired with Vγ4+ in the human colon	*GZMB, PRF1, IFNG*; state: *IL23R*	Mostly anti-tumoral (cytotoxic); Pro-tumoral (AREG producing)	(21, 24–30)
Vδ3	Regulatory/immunomodulatory; IL-17-capable	Mucosa	CD56^+^, CD161^+^	*RORC, IL23R*	Pro-tumoral (understudied)	(15, 21, 25, 27, 31)

The table summarizes major γδ T cell populations in mouse and human colon, highlighting tissue localization, key surface markers and transcripts, primary biological functions, and roles in cancer. Mouse lamina propria γδT17 cells (Vγ4/Vγ6) are IL-17–producing, microbiota-dependent, and contribute to tissue repair and pro-tumoral processes such as angiogenesis and myeloid recruitment. Intestinal intraepithelial lymphocyte (IEL)-type subsets (Vγ7/Vγ1) are cytotoxic, granzyme-rich, and support epithelial surveillance. Human circulating and tissue-resident subsets (Vγ9Vδ2, Vδ1, Vδ3) display distinct effector or regulatory phenotypes, contributing to anti-tumoral cytotoxicity or context-dependent pro-tumoral immunoregulation.

The presence of a gut-intrinsic γδ T-cell subset appears to be conserved across evolution. In humans, intestinal γδ IELs are predominantly composed of cells expressing a Vδ1 TCR chain paired most commonly with Vγ4. These γδ IELs typically express CD103, reflecting their tissue-resident epithelial localization. Similar to the mouse system, human γδ IELs are shaped by BTNL molecules: specifically, BTNL3 and BTNL8 form a heterodimer that drives the selection, maturation, and maintenance of Vγ4^+^ Vδ1^+^ IELs (26). A key difference in humans, however, is that Vγ4^+^ γδ IELs do not strictly dominate the γδ T cell compartment. Instead, they coexist with additional clonotypes, including Vγ2/3^+^ Vδ1^+^ populations, contributing to greater repertoire diversity compared with murine γδ IELs (29). What this signifies for humans is not completely understood. The functional importance of BTNL-dependent selection becomes particularly evident in pathological settings. For example, in celiac disease, epithelial expression of BTNL3/8 is markedly diminished. Correspondingly, the Vγ4^+^ Vδ1^+^ IEL subset that relies on BTNL signaling is drastically reduced, highlighting both their dependence on epithelial BTNL expression and the impact of epithelial stress or inflammation on γδ IEL homeostasis (28).

Beyond their distinctive TCRs, γδ IELs rely on conserved adhesion and regulatory receptors that support their epithelial residency and rapid surveillance functions in both mice and humans. They continuously “floss” between enterocytes and patrol the basement membrane, a behavior enabling early detection of epithelial stress or breaches (33, 34). CD103 engages E-cadherin to anchor them within the epithelial layer and stabilize IEL–enterocyte contacts, thereby coordinating retention with focused effector delivery (35, 36). To prevent excessive activation in a stimulus-rich environment, γδ IELs express CD8αα, which binds TL antigen on enterocytes to attenuate TCR signaling and maintain a poised, high-threshold activation state (20, 37, 38). Upon epithelial stress, activating NKRs such as NKG2D override this restraint and rapidly induce cytotoxic programs including granzymes, perforin, FasL and TNF-α, allowing elimination of damaged or infected enterocytes. Cytokine cues such as type I interferons can further potentiate antimicrobial functions including IFN-γ production (32, 39–41).

### 1.4 γδ lamina propria lymphocytes

Below the basal membrane, in the lamina propria, resides another distinctive set of γδ T cells, which represent a rather different “flavor” of the γδ T cell compartment. In mice, this population is enriched for IL-17-producing clonotypes that often express a Vγ4^+^ or Vγ6^+^ TCR, instead of the Vγ7^+^ subset that dominates the epithelial layer. These γδ LPLs are functionally biased toward RORγt-driven IL-17/IL-22 programs, rather than the cytotoxic programs that IELs display (42). In humans, this population is more heterogeneous. Vδ1^+^ cells dominate the tissue-resident human gut γδ T cell compartment, which they share with Vδ2^+^ and Vδ3^+^ cells, but the distinction between clonotypes that inhabit the IEL vs. the LPL compartment is blurrier than in the mice colon. Their phenotype of human γδ LPLs is also a matter of discussion, as they seem not to be completely driven towards the production of IL-17 like their murine counterparts, but to be mostly cytotoxic (43).

Unlike γδ IELs, these lamina propria γδ T cells do not rely on BTNL-driven selection or survival signals and thus are not shaped by BTNL1/6 (or BTNL3/8 in humans). Their maintenance and activation are instead tied to a completely different set of cues (9). In mice, it was shown that these cells respond to a combination of innate cytokines and microbial products, often with minimal requirement for classical TCR triggering (44, 45). These cues include barrier-associated microbial signals that promote IL-23 and IL-1β production, as well as metabolic and environmental mediators such as AHR ligands, including the tryptophan-derived metabolite FICZ, which together promote RORγt and shape the IL-17/IL-22-skewed functional profile characteristic of Vγ4^+^ and Vγ6^+^ LP γδ T cells (46, 47). Coherently, antibiotic treatment or alterations of the microbiota modulate the frequency and IL-17 output of these cells in the cecum and colon, pointing towards this microbiota-cytokine axis being a dominant force in the differentiation and maintenance of γδ17 LPLs (44).

Functionally, γδ LPLs are tissue-resident, but distinct from epithelial IELs. While expressing activation or residency markers such as CD69 and CD103 variably, they do not express CD8αα. Furthermore, many express CCR6, which attracts them to CCL20 and may facilitate the recruitment of these cells to the lamina propria. Their expression of RORγt, IL-23R and IL-1R are also hallmarks of lamina propria γδ T cells that enable rapid cytokine responses and distinguishes them from IELs (13).

Lamina propria γδ T cells are a major innate source of IL-17 and IL-22 at steady state and during early responses to infection or epithelial damage. IL-17 production promotes neutrophil recruitment and antimicrobial peptide expression; IL-22 supports epithelial repair and barrier function. In mouse models, γδ17 LPLs are protective in some acute damage models (e.g., DSS colitis, epithelial injury) but can also contribute to pathology in chronic inflammatory settings depending on context and cytokine milieu. Their cytokine output is rapidly potentiated by IL-1β/IL-23 and can be restrained by regulatory signals in the tissue microenvironment (42, 48).

## 2 γδ T cells in CRC

The γδ T cell compartment in colorectal cancer (CRC) is highly plastic and shaped by the specific tumor milieu, with marked heterogeneity in density, subset composition, spatial distribution, and functional state across tumor subtypes (**Table 1**). Importantly, γδ T cells do not uniformly infiltrate all colorectal tumors. In gut tumors driven by activating WNT-pathway mutations (as in the majority of sporadic CRC), γδ T cells can be effectively excluded from the epithelial compartment, likely due to dysfunctional WNT signaling and consequent loss of BTNL expression by transformed epithelial cells, which disrupts patrolling by γδ intraepithelial lymphocytes (49). By contrast, colitis-associated cancer (CAC) models such as AOM/DSS—less dependent on WNT alterations—are more permissive to γδ T-cell infiltration (15, 50), underscoring that epithelial signaling programs critically gate γδ T-cell access.

Within tumors that do permit infiltration, γδ T cells exhibit distinct and non-random spatial organization. Multiplex imaging and high-dimensional *in situ* proteomics demonstrate that both Vδ1 and Vδ2 cells can be found in direct contact with tumor cells, but they occupy partially distinct niches: Vδ1 cells are often enriched near the tumor–endothelial interface and show clonal sharing with peripheral blood, consistent with active trafficking between circulation and tumor, whereas Vδ2 cells display greater functional heterogeneity within the tumor parenchyma (51). Quantitative spatial analyses further reveal that γδ T cells are, on average, located ~25% closer to malignant epithelial cells than conventional αβ T cells, suggesting preferential positioning for direct tumor interaction (52). Nevertheless, their localization varies across the tumor landscape (tumor core vs invasive margin vs stroma), indicating that meaningful interpretation requires spatial analysis.

γδ T-cell density and positioning are also tightly linked to tumor molecular features and clinical behavior. Across large patient cohorts, higher γδ T-cell densities associate with lower disease stage, higher tumor grade, reduced lymphovascular invasion, mismatch-repair deficiency, and BRAF mutation, and, when either αβ or γδ T cells are abundant, correlate with improved cancer-specific and overall survival (52). Notably, γδ T cells are relatively enriched in tumors with defective antigen presentation, including β2-microglobulin (B2M) loss, independent of MMR status, consistent with their capacity for MHC-independent tumor recognition (21, 53). Single-cell analyses likewise showed that γδ T cells preferentially accumulate in tumors with weak antigen-presentation signaling pathways, aligning with a role for γδ T cells in immune surveillance when classical CD8^+^ T-cell recognition is compromised (52).

Functionally, tumor-infiltrating γδ T cells in CRC are heterogeneous and context-dependent. In human CRC, Vδ1 cells are prevalent but often display a dysfunctional, yet not fully exhausted, state, characterized by altered inhibitory and activating receptor expression and reduced cytotoxic effector programs (including lower IFNG and GNLY expression in several cohorts), particularly in MSS tumors (54). These alterations appear at least partly shaped by stromal interactions (e.g., fibroblast TIGIT–NECTIN2 signaling). In contrast, Vδ2 cells show greater proliferative activity within CRC (higher MKI67 expression) compared with healthy colon, suggesting differential subset dynamics during tumor progression. Overall, γδ T cells are generally less abundant in CRC than in healthy colon, implying selective exclusion, reprogramming, or loss of tissue-resident pools during malignant transformation.

These spatial and functional differences highlight the ambivalent potential of γδ T cells in cancer: depending on context, they may contribute to anti-tumor immunity, promote inflammation and tumor progression, or be largely excluded from the tumor microenvironment. Understanding these nuances is essential for leveraging γδ T cells in immunotherapy, as their therapeutic utility will depend on tumor subtype, microenvironmental signals, and the functional state of resident γδ subsets.

Finally, several apparent discrepancies between murine and human γδ T-cell biology, particularly regarding IL-17–producing γδ T cells, reflect both true species differences and methodological hurdles. Murine γδ17 cells (mainly Vγ4^+^/Vγ6^+^) are developmentally pre-programmed for IL-17 production via thymic imprinting and strong RORγt dependence (47, 55, 56). In contrast, human gut γδ T cells, dominated by Vδ1^+^ and Vδ2^+^ subsets, lack this intrinsic type-3 bias and are primarily cytotoxic (24, 29, 57). Moreover, technical limitations complicate human data: scRNA-seq frequently mis-annotates IL-17–expressing αβ T cells as γδ T cells due to low TCRD coverage, variable-region dropout, and mixed-chain assignment (57), while flow-cytometric identification varies widely in gating strategies and detection of Vδ3^+^ cells (29). Considering these issues, bona fide γδT17 cells appear rare or absent in the steady-state human gut, in stark contrast to murine models where γδ17 cells are abundant and dominant during tumorigenesis. In human CRC, IL-17–producing γδ T cells thus remain highly context- and dataset-dependent (15, 24, 27, 47, 57).

### 2.1 Pro-tumoral functions of γδ T cells: are human γδ T cells capable of tumor promotion?

Descriptions of γδ T cells assuming a pro-tumoral role in various experimental tumor models are not scarce. In ovarian and breast cancer models, these cells promote tumors by stimulating the angiogenic switch and recruiting immunosuppressive myeloid cells into the tumor microenvironment (14, 58). Similar mechanisms are in place in fibrosarcoma models, where TCR- and NKG2D-mediated IL-17 production by γδ T cells is responsible for tumor promotion via stimulation of angiogenesis (59). Furthermore, implanting breast, hepatocellular, and melanoma cancer cell lines in IL-17KO mice resulted in reduced tumor growth (60–63).

**Figure 1:**
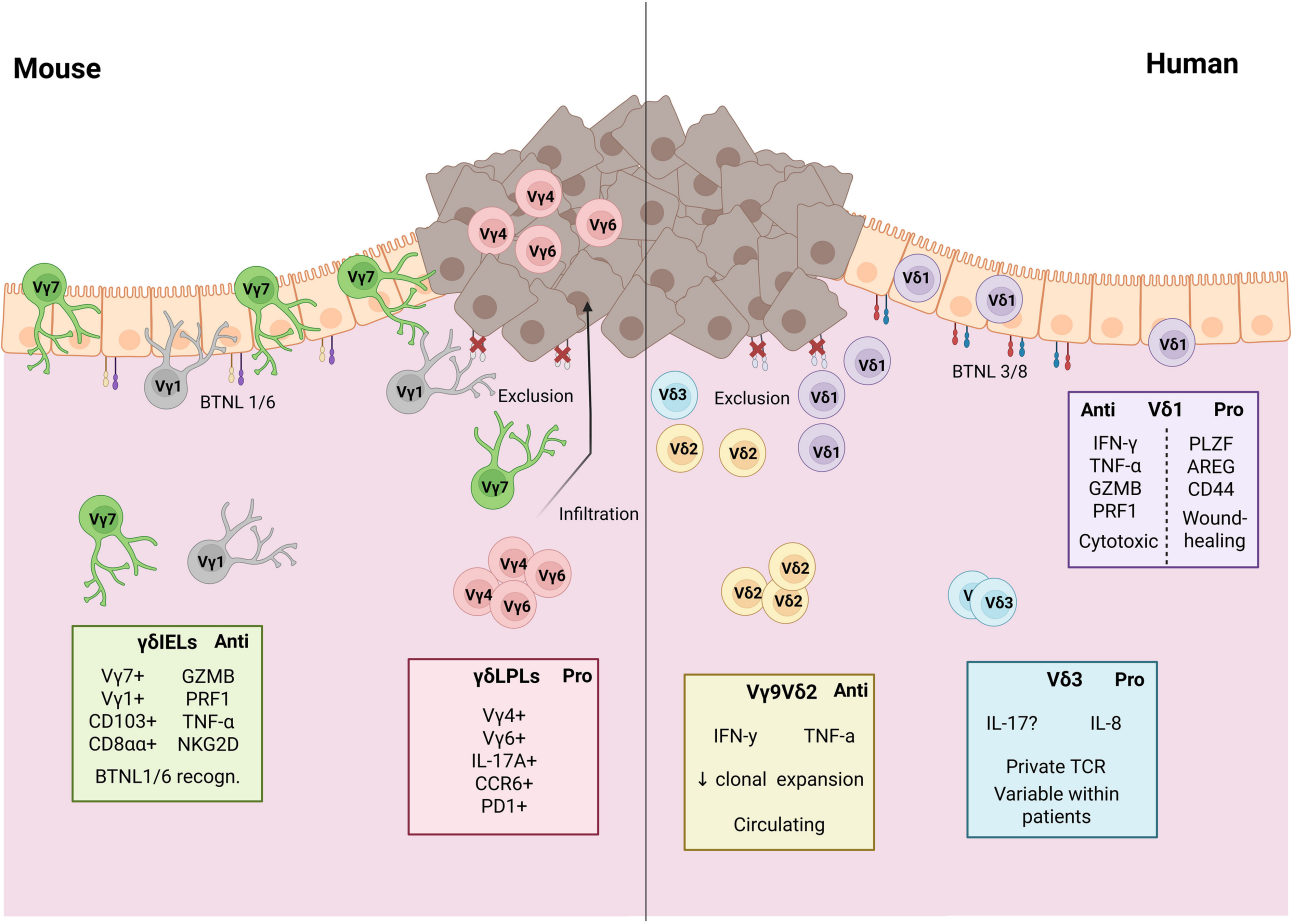
Distinct γδ T-cell subsets in colorectal cancer (CRC) in mice and humans. (Left) – Mouse CRC. In the colon, intraepithelial γδ T cells (γδ IELs) are dominated by Vγ7^+^ and Vγ1^+^ subsets that recognize epithelial BTNL1/6 and exhibit an anti-tumoral cytotoxic phenotype, including expression of granzyme B, perforin-1, TNF-α, NKG2D, CD103, and CD8αα. During tumor development, loss of BTNL expression on transformed epithelial cells leads to exclusion of γδ IELs from the tumor tissue. In contrast, lamina propria γδ T cells (γδ LPLs), primarily Vγ4^+^ and Vγ6^+^, infiltrate CRC lesions and display a pro-tumoral profile, characterized by IL-17A, PD-1 and CCR6. (Right) – Human CRC. Human colorectal tissue also contains heterogeneous γδ T-cell subsets with both anti- and pro-tumoral properties. Vδ1^+^ γδ T cells usually adopt an anti-tumoral cytotoxic program (producing IFN-γ, TNF-α, granzyme B, and perforin-1) but are also reported to display a pro-tumoral, tissue-repair–associated phenotype, marked by PLZF, AREG, and CD44 expression, in rarer cases. Circulating Vγ9Vδ2 T cells, which show limited clonal expansion within tumors, are predominantly anti-tumoral and produce IFN-γ and TNF-α. Emerging Vδ3^+^ populations show variable expansion across patients, harbor private TCR repertoires, and may exhibit pro-tumoral cytokine profiles including IL-8 and IL-17, though their roles in CRC require further clarification. Created with BioRender.com.

Recent findings by Reis and colleagues showed that CAC models further support this rationale: in AOM/DSS-treated mice, tumors are infiltrated by Vγ4^+^ and Vγ6^+^ γδ T-cell populations that closely mirror each other phenotypically (**Figure 1**). These differ markedly from γδIELs as they lack CD8αα expression and instead express PD-1. Upon stimulation, LPL Vγ4^+^ and Vγ6^+^ γδ T cells become the predominant source of IL-17A within the tumor tissue (40% of IL-17A producing cells are γδ T cells). This finding was further confirmed in the same study using a CRC model driven by loss of the tumor suppressor gene APC, where γδ T cells are 60% of all IL-17 producers in the tumor. This dominant phenotype within the tumoral milieu seems to be led by an increase in *in situ* proliferation of these cells, rather than an exclusion of CD4^^+^^ αβ T cells from the tumors. This *in-situ* proliferation is caused by changes in the microbiota compartment induced by carcinogenesis in the APC loss mouse model (15). Another earlier study also associated CRC with the presence of IL-17-producing γδ T cells in mice; however, in this murine model, γδ T cells functions were redundant to those of the αβ T cells (64).

However, this still leaves the question open whether γδT17 cells are promoting tumoral growth solely via IL-17 secretion or also by other mechanisms. In the current literature, there are several descriptions of pro-tumoral activity in γδ T cells that infiltrate CRC tumors. Wu and colleagues proposed that IL-17 production in human CRC was due to CD3^+^ cells upon re-stimulation, and that within the CD3^+^ cell compartment, γδ T cells were the main IL-17-producing subset (65). Aside from IL-17, these cells also produced TNF-α and, in contrast to Th17 cells, never produced IFN-γ, like it may have been expected. In this work, the authors also describe how these cells are primed to produce high levels of IL-17 by tumor infiltrating dendritic cells, which express IL-23 in a microbiota dependent manner. These tumor-infiltrating (TI) γδ17 cells were also shown to produce TNF-α, IL-8 and GM-CSF, which are linked to the accumulation of polymorphonuclear-myeloid derived suppressor cells in the tumor (65). This link has already been established in some mouse tumor models, like in ovarian cancer and breast cancer, where γδ T cells are shown to promote the accumulation of macrophages and neutrophils, respectively (14, 58).

Along the same line, (27) provided evidence that the γδ T cell infiltrate in human CRC is not uniformly anti-tumoral, and the more heterogeneous, the worse the outcome in CRC. Their work delineates a cytotoxic γδ program alongside a PLZF^+^, AREG-producing Vδ1 subset with pro-tumor, tissue-repair-like properties, that was able to promote CRC proliferation during *in vitro* scratch assays. Furthermore, they identified a Vδ3^+^ IL-17A-producing population within the γδ compartment. These findings reinforce a model in which γδ T cells harbor multiple transcriptional and functional states, some of which may actively support tumor progression. The idea of a putative Vδ3^+^ subset that is responsible for IL-17 production is further corroborated by the work of (25), who demonstrated that a Vδ3^+^ subset is clonally expanded in CRC and has tumor-specific, private TCR repertoires. In general, IL-17 production was overall low in the γδ T cell population, but attributed mainly to Vδ3^+^ γδ T cells.

In the other hand, a recent meta-analysis by Ran and colleagues challenged the notion that IL-17-producing γδ T cells constitute a substantial population in human CRC. First, the authors showed that clusters previously annotated as γδ17 cells in the work of Reis and colleagues exhibit very low levels of IL17A and RORC transcripts, making a real γδ17 identity unlikely (15, 57). Moreover, the only cluster with detectable IL17A and RORC expression displayed a T-helper-like transcriptional profile, including low but measurable CD4 expression and an absence of TCRD transcripts, indicating that it is more consistent with an αβ T-cell population than with γδ T cells. Then, using a stringent, lineage-defining gamma delta T cell signature, the authors analyzed more than 18000 γδ T cells from neoplastic and adjacent healthy tissue of 165 CRC patients and found that human tumor-infiltrating γδ T cells are predominantly associated with anti-tumor rather than IL-17-driven pro-tumor programs. Across nine independent datasets, expression of key IL-17-associated transcription factors (RUNX1, IRF4, BATF, KLF4, STAT3) was sparse or inconsistent, and RORC, which is required for IL-17 production, was essentially absent, further supporting the limited IL-17 potential of human γδ T cells in CRC (57). Supporting these findings, γδ T cells were described as not being among the main contributors of IL17A expression in CRC tumors from over 1600 patients (52).

These observations further support the work by Meraviglia and colleagues, who showed that the majority of IL17^+^ cells in the tumor did not express either of the VD chains, suggesting they might be indeed Th17 or Tc17 cells. Additionally, the authors demonstrated that, in CRC transcriptomic datasets, the expression of γδ TCR-related genes TRGV9, TRDV2 and CD3D correlated with IFNG expression but not IL17A, further suggesting that γδ T cells in the context of human CRC are of the cytotoxic, IFN-γ producing phenotype. In sum, while there are multiple strands of evidence pointing toward a pro-tumoral γδ17 program in mice, especially in settings where the general presence of microbiota and local inflammation drive *in-situ* expansion of IL-17-producing Vγ4^+^ and Vγ6^+^ cells (15), the data in humans is more complex. The work by Wu et al. clearly describes γδ T cells as major IL-17 producers in human CRC (65), shaping the myeloid landscape and supporting tumor growth, while others reported that a small subset of Vδ3^+^ γδ T cells produces IL-17 in the tumor, but not in the levels of Th17 cells (25, 27). In some cases, Vδ1^+^ γδ T cells also promote tumor progression through wound-healing or tissue-repair-like programs such as AREG production (27). In contrast, other studies argue that γδ17 cells are essentially absent from human CRC tissue and that tumor-infiltrating γδ T cells are predominantly cytotoxic, IFN-γ-producing populations with very limited IL-17 potential (24, 57). These contradictions imply that γδ T-cell behavior in human CRC may be far more sensitive to context than what is depicted in mice, perhaps influenced by microenvironmental cues and the technical methods used to identify these cells.

### 2.2 Anti-tumor functions of γδ T cells

γδ T cells are often described in the literature to exert anti-tumoral functions across different cancer types, including prostate cancer, spontaneous B-cell lymphoma and several skin cancer subsets (63). These observations align with multiple CRC murine models in which γδ T cells display potent anti-tumoral activity. In carcinogen-induced CRC (AOM/DSS) and APC-loss–driven models, constitutive TCRδ-knockout mice consistently develop a significantly higher tumor burden than γδ-sufficient controls, suggesting an essential role for γδ T cells in early tumor surveillance (15, 66–68). However, because these studies rely on constitutive knockout systems, the interpretation of γδ-T-cell–mediated effects is inherently complicated. Lifelong absence of γδ T cells reshapes multiple developmental programs, both immune and epithelial, that can obscure immediate γδ-dependent functions.

On the immune side, several studies have shown that loss of γδ T cells triggers compensatory expansion or functional skewing of other lymphocyte compartments, including increased αβ T-cell activation and NK-cell compensation (69). These shifts can partially mask or substitute for γδ-mediated surveillance, making it difficult to define the direct contribution of γδ T cells to tumor control.

Equally important, γδ T cells imprint epithelial and stromal programs that are altered in their absence. Seminal work in skin and intestinal epithelia has shown that γδ T cells shape epithelial repair, tight-junction stability, and stress-surveillance circuits through factors such as keratinocyte growth factor (KGF), IL-22, and junctional cues (70–72). γδ-deficient mice demonstrate aberrant epithelial proliferation, impaired wound repair, and altered stromal cytokine landscapes—all of which influence how tumors emerge or progress within these tissues. As a result, constitutive TCRδ-knockout models may underestimate the true scope of γδ-T-cell contributions to CRC biology, because both immune compensation by other lymphocytes (“wannabe γδ T cells) and epithelial/stromal mis-patterning accumulate from early ontogeny onward. Approaches using inducible γδ-cell depletion or adoptive transfer would better isolate the acute, context-dependent roles of γδ T cells. Mechanistically, the protective activity of γδ T cells in CRC models is attributed to the IEL compartment, rather than to the LPLs. Interestingly, Vγ1^+^ and Vγ7^+^ actually seem to be redundant in function, as both need to be depleted in order to abrogate tumor control. This is particularly interesting, given the different ontogeny and phenotype of these two subsets (**Figure 1**) (15, 43). These anti-tumoral subsets are shown to have a glycolytic-dependent metabolism, as Glut1 deletion in γδ T cells leads to a loss of tumor control, which is in line with descriptions in the literature of γδIFN cells relying on glycolysis (15, 73). Coherently, the deletion of Glut1 was more preponderant in an earlier stage of carcinogenesis, contributing to the idea of γδ T cells being early sources of cytotoxicity in the context of cancer (74). Notably, despite their protective potential, IELs seem to accumulate in the tumor-adjacent region, which implies that the anti-tumoral function of these cells might be severely impaired after tumor establishment. While this reduced activity is partially microenvironment-driven, intrinsic transcriptional restraint, particularly mediated by TCF-1, further limits their effector program. TCF-1 deletion unleashes IELs, enhancing their tumor-suppressive activity and driving higher expression of cytotoxic and effector molecules, including GZMA, GZMB, IL2RB, TNFRSF9, and XCL1. (67).

Human datasets are broadly consistent with this anti-tumoral narrative. γδ T cells preferentially accumulate not only in MMRd tumors, but also in CRCs bearing BRAF, TP53 and B2M mutations (21, 24, 52). Only in B2M mutated tumors is this accumulation distinct from αβ T cells, likely because γδ T cells are not dependent on classical antigen presentation, which allows them to perform in environments where HLA expression has been downregulated, a regular occurrence during the process of carcinogenesis (75, 76). Although they comprise around 20% of tumor infiltrating CD3^+^ cells, γδ T cells are mainly detected in the stroma or border of the tumor, very rarely invading the tumor (24). This mirrors murine IEL behavior, and is likely driven by disruptions of the WNT pathway signaling during the process of carcinogenesis. The findings by (49) underscore that BTNL downregulation in WNT-driven CRC is not a passive consequence of oncogenic signaling but a change with direct impact on γδ T-cell exclusion. Human datasets point in the same direction: BTNL3 and BTNL8 are consistently reduced in colorectal tumors relative to normal tissue, and their expression positively correlates with γδ T-cell infiltration (49, 77). These data strengthen the idea that the epithelial BTNL program is a key determinant of γδ IEL access across species. Whether partial WNT-pathway inhibition or restoration of epithelial differentiation could re-establish BTNL expression in WNT-addicted tumors remains completely unknown, but the preserved γδ infiltration in CAC and in non–WNT-driven human CRC subtypes indicates that this axis is not uniformly lost. Identifying which molecular subtypes retain an intact BTNL program will be critical for understanding when γδ IEL surveillance can be recovered and when exclusion is likely irreversible. Importantly, their inability to penetrate the tumor does not necessarily hinder the anti-tumor function of these cells, as higher numbers in tumors correlates with longer 5-year disease free survival, as well as prolonged overall survival in CRC patients (24, 52, 78).

Among human subsets, Vδ1^+^ γδ T cells are the dominant tumor-resident population in CRC (21). Functionally, this subset is heterogeneous. On the one hand, Vδ1^+^ cells repeatedly cluster with potent anti-tumoral programs characterized by high GZMB, PRF1, and IFNG expression (24, 25). This is consistent with the findings of Mikulak et al., which demonstrate that NKp46 expression defines the largest Vδ1^+^ subset, which harbors high cytolytic potential, as well as the capacity for IFN-γ production. Furthermore, this subset preferentially pairs the Vδ1 with a Vγ4 TCR chain, pointing towards a natural IEL ontogeny (29). On the other hand, a minor PLZF^+^, Amphiregulin-producing Vδ1 subset described earlier displays wound-healing features with potential pro-tumoral roles (27). Despite this duality, the bulk of Vδ1^+^ cells in human CRC align with an anti-tumoral, cytotoxic phenotype, which is consistent across scRNA-seq datasets.

Vδ2^+^ cells, although more abundant in blood, remain substantially represented within tumor tissue (24, 78). Although canonical Vγ9Vδ2 T cells can adopt an IL23R^+^ RORG^+^ type-3 immunity phenotype (79, 80), they are consistently described as strongly anti-tumoral, exhibiting high IFN-γ and TNF-α production and robust cytotoxicity *in vitro* and ex vivo. Vδ2^+^ cells constitute the majority of IFN-γ^+^ and TNF-α^+^ γδ T cells in CRC tumors (24, 25). Thus, while both Vδ1 and Vδ2 subsets can contribute to antitumor immunity, Vδ2^+^ cells are the most uniformly anti-tumoral subset across human studies.

Besides the two main subsets, the data on the smaller Vδ3^+^ subset is rather inconclusive. Some studies report Vδ3^+^ cells with capacity of expressing cytotoxic cytokines and stress-responsive transcriptional signatures in tumor adjacent tissues (15, 25). Contrastingly, other studies suggest that they might be involved in tumor progression, as described in the section before (25, 27). Altogether, the data on this subset suggests functional heterogeneity, mirroring the duality of Vδ1^+^ γδ T cells.

Together, while the data in mice shows a split reality between pro- and anti-tumoral functions, depending on context, the results from human cohort studies paint a clearer picture: the predominant functional identity of γδ T cells in human CRC is anti-tumoral, cytotoxic, and IFN-γ-biased, with Vδ1^+^ and Vδ2^+^ populations forming the principal effector subsets (15, 24). Although their function can be impaired regarding the inability to penetrate the tumor borders or being affected by the tumor secretome, these cells’ functions remain consistent (24). Despite their restricted infiltration into tumor nests, higher γδ T cell presence remains robustly associated with improved patient outcomes, underscoring their relevance as endogenous mediators of tumor control in human CRC (24, 52, 78).

## 3 Therapeutic implications and concluding remarks

The data in mouse models send a clear and structured message: γδ T cells are, as a whole, protective in the context of CRC. Several independent studies using TCRdKO mice in different CRC models report higher tumor burden in mice that are deficient in γδ T cells (15, 66–68). However, when looking deeply into the quality of these cells, the picture is more complex. These cells are composed by heterogeneous subsets: an anti-tumoral one, led by the by Vγ1^+^ and Vγ7^+^ IELs, which are cytotoxic and prefer a glycolytic metabolism; and a pro-tumoral one, which is formed by LPLs expressing the Vγ4 or Vγ6 TCR chain, capable of infiltrating the tumor and of producing IL-17 (15, 68). However, some topics remain to be clarified in the context of mouse CRC models. Firstly, what licenses some γδ T cell subsets to efficiently infiltrate the tumors, while others are stuck in the stroma and bordering tissue? Secondly, what are the mechanisms behind γδ T cell-led tumor promotion? Furthermore, would there be a difference between constitutive absence of γδ T cells and inducible depletion of these cells? And lastly, how would different kinds of microbiome modulate the action of anti-tumoral and pro-tumoral γδ T cells in CRC models? In humans, the picture is even less clear-cut than in mice, but the overall trend strongly supports a generally protective role for γδ T cells in colorectal cancer. Multiple studies show that higher γδ T-cell infiltration correlates with improved disease-free and overall survival, indicating that these cells are generally beneficial in the human setting (24, 52, 78). Some reports further show that cytotoxic, NKp46^+^ γδ T cells are enriched in lower-grade tumors and markedly reduced in higher-grade ones (29). However, based on the current data, it remains difficult to determine whether this pattern reflects an active role for γδ T cells in restraining tumor progression or whether carcinogenesis progressively excludes or disables these cells within the tumor microenvironment, as proposed by Suzuki and colleagues (49).

Is there also evidence for pro-tumoral effects of γδ T cells in human CRC? One prominent description of a major IL-17 producing γδ T cell subset in human CRC (65) was countered by other studies, which failed to reproduce the same results when using an optimized gating strategy and gene signature that efficiently excludes αβ T cells and NK cells in their entirety (24, 57);. Harmon and colleagues proposed that there is a pro-tumoral Vδ1^+^ population that is independent of the expression of IL-17, but there is still a lack of *in vivo* data that corroborates this hypothesis (27).

The intra-subset heterogeneity of γδ T cells observed in human data highlights the necessity of better characterization of the expanded clones. Few publications are reporting on the TCR chains of expanded clones. Reis and colleagues reported that Vδ1Vγ4 are expanded both in tumor-adjacent and intra-tumoral areas, but with opposing phenotypes (cytotoxic versus IL17-producing, respectively). They also found a Vδ3Vγ8 subset expanding in the tumor-adjacent area, which is deemed cytotoxic/anti-tumoral (15). Surprisingly, this contrasts with other studies that describe Vδ3 cells as mainly pro-tumoral (25, 27). Altogether, these findings do not yet provide sufficiently robust evidence to link certain clonotypes or Vγ/Vδ usage to pro- or anti-tumoral roles. This question is of great relevance, as knowledge about the specific clones that are beneficial could contribute to more efficient anti-CRC therapies in the long run.

Little is known about what drives the recognition of CRC tumors by γδ T cells. The presence of specific TCR motifs in gut-intrinsic γδ T cells suggests that the TCR plays an important role in recognizing a ligand that drives the killing of transformed cells. For example, circulating Vγ9Vδ2^+^ γδ T cells recognize phosphoantigens expressed in stressed cells (23). Furthermore, there is evidence that the activating NK receptors in these cells are also involved in the killing of these tumoral cells. Blocking of NKG2D leads to both reduced activation and killing of the same cells (21). The importance of NKG2D for tumor recognition remains true even in the case of DOT cells in CRC, which is coherent to what was shown in liquid tumors. On the other hand, blocking of the TCR of Vδ1^^+^^ and Vδ3^+^ cells during co-culture with CRC organoids leads to unchanged activation and killing of the malignant cells (21). This is specially surprising given how important the TCR of Vγ9Vδ2 γδ T cells is for tumor recognition, and raises the question whether gamma delta TCR signaling can be effectively blocked by using antibodies (22).

While the data in humans was mostly of an observational nature, recent studies have tried to demonstrate that not only γδ T cells correlate with better outcomes, but also can target and kill tumor cells. De Vries and colleagues demonstrate this *in vivo*, where Vδ1+ and Vδ3+ γδ T cells exerted killing in different CRC cell lines and organoids, with preferential reactivity towards the HLA class I negative ones (21). Harmon and colleagues were able to halt tumor growth in humanized mice bearing patient-derived xenografts by transferring expanded Vδ1 ^+^ cells with cytokines that revert their original pro-tumoral behavior, and shape them towards an anti-tumor phenotype (27). Importantly, it needs to be determined whether these data reflect the plasticity of the subset, or whether the authors’ protocol favored the expansion of an anti-tumoral sub-clone. In the same direction, Blanco-Dominguez et al. used the already established DOT (Delta One T) cells and demonstrated that their efficacy goes beyond liquid cancers, as expanded DOT cells target and kill CRC both *in vitro*, but also *in vivo*, in mice expressing hIL-15, which is necessary for the maintenance of these cells (81).

Recent work now allows a more concrete evaluation of γδ T cells as therapeutic agents in CRC, moving beyond earlier conceptual proposals. Several γδ T-cell–based modalities are under active preclinical and early clinical development, each facing distinct opportunities and limitations.

Current γδ T-cell immunotherapy efforts largely rely on Vγ9Vδ2 cells. These cells can be activated *in vivo* by phosphoantigens or expanded ex vivo using zoledronic acid prior to adoptive transfer. A major limitation, however, is that phosphoantigen stimulation is not durable: repeated exposure drives Vγ9Vδ2 cells toward functional exhaustion, reducing their therapeutic potential (82). Vδ1-based Delta One T (DOT) therapy is also a promising candidate in clinical trials, which involves expansion of peripheral Vδ1 cells ex vivo through TCR- and cytokine-mediated stimulation to induce NCR expression and robust cytotoxicity. DOT cells kill both MSI-H and MSS CRC organoids and cell lines, and their activity is further enhanced by combined PD-1 and TIGIT blockade (27, 81). DOT-cell efficacy *in vivo* requires IL-15 supplementation to sustain the transferred cells (81).

High-resolution profiling has now shown that PD-1^+^ γδ T cells, specially Vδ1 and Vδ3 cells, accumulate in MSI-H CRC and act as major effectors of PD-1 blockade in the context of HLA class I loss (21, 53). In these tumors, PD-1^+^ γδ T cells exhibit proliferative and cytotoxic signatures and mediate killing of HLA-I–deficient organoids, providing a mechanistic explanation for why patients with B2M mutations respond strongly to anti-PD-1 therapy. In MSS CRC, γδ T-cell dysfunction is more prominent: Vδ1 cells display a TIGIT-dependent suppressive program driven by fibroblast-expressed NECTIN ligands and can be partially rescued by TIGIT blockade (54). TIM-3 also impairs Vγ9Vδ2 cytotoxicity against CRC (83). These findings support the rationale for combination checkpoint strategies tailored to specific γδ subsets, an approach also highlighted by recent reviews (84).

Three major challenges still consistently emerge across platforms. (1) Persistence: γδ T-cell products, particularly Vγ9Vδ2 cells, exhibit short-lived survival in solid tumors without cytokine support; IL-15 or metabolic enhancement improves persistence, at least on their Vδ1 counterparts, but may raise safety concerns (81). (2) Trafficking: stromal remodeling and chemokine mismatches in CRC limit γδ T-cell infiltration, consistent with findings that many Vδ1 cells remain peritumoral rather than intraepithelial (84). (3) Safety: although γδ T cells carry low GVHD risk due to MHC-independent recognition, their dependence on NKG2D and other stress ligands raises concerns for on-target activity in inflamed epithelia. Early CAR-γδ trials have therefore proceeded conservatively, with limited but encouraging safety signals to date (85).

γδ T cells are now established characters in the landscape of CRC and therefore it is commendable that academic and translational attention is shifting towards this less conventional subset of T cells. Many questions are still to be answered: how γδ T cells recognize CRC in a TCR-dependent or TCR-independent manner? Which ligands promote activation of pro- and anti- tumoral subsets? To what extent does the microbiome influence their function? And does the pro-tumoral function of γδ T cells derive from intra-subset plasticity, or merely expansion of already programmed clones? Answering these questions is fundamental for the future manipulation of γδ T cells in the context of CRC immunotherapy.
